# Giant Pilomatrixoma Presenting in the Posterior Thorax, a Rare Location and the Largest Described

**DOI:** 10.1155/2015/590742

**Published:** 2015-02-11

**Authors:** P. Gongidi, J. Meshekow, T. Holdbrook, P. Germaine

**Affiliations:** ^1^Department of Radiology, Cooper University Hospital, Cooper Medical School of Rowan University, Camden, NJ, USA; ^2^Department of Pathology, Cooper University Hospital, Cooper Medical School of Rowan University, Camden, NJ, USA

## Abstract

Pilomatrixoma is a common benign soft tissue neoplasm arising from hair follicle cells, typically not exceeding 3 cm and located mainly within the head and neck regions. Lesions greater than 3 cm or those located elsewhere are rare and are often not thought of or high on a differential diagnosis. Moreover, the radiographic features of pilomatrixoma are very nonspecific making the diagnosis even more difficult and rarely described in the radiology literature. We present the largest reported case of pilomatrixoma measuring 24 cm arising from the posterior thorax. Our hope is to increase awareness of this diagnosis for slow-growing soft tissue masses not located in the classically described locations of head and neck, explore the radiographic features on various imaging modalities, and review the current radiology literature.

## 1. Case Report

**Figure 1:**
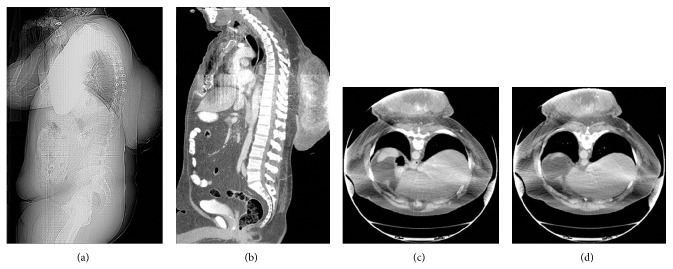
The scoutogram (a) reveals a very large bulky mass within the posterior midback. Sagittal (b) and axial contrast enhanced CT images ((c) and (d)) show a large heterogeneous mass which is not completely imaged and partially extrinsically compressed in the gantry. The mass demonstrates central area of hypodensity consistent with necrosis as well as scattered, coarse calcifications. This mass is in close proximity to the superficial skin and subcutaneous tissue without much involvement of deep structures.

**Figure 2:**
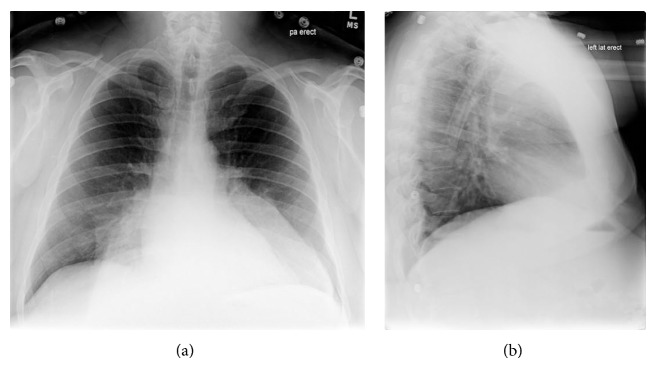
The PA (a) radiograph of the chest reveals a double-density opacity overlying the cardiac silhouette which on lateral (b) radiograph does not appear to localize to the anterior thorax or any compartment of the mediastinum.

We present a 52-year-old gentleman with a large mass in the upper back, which originally started as an “itchy bump” 9 years ago and has progressively doubled in size each year. He presented to the urgent care clinic because of bleeding and purulent discharge from the mass and subsequently he was referred to the emergency department for further workup. Lab results showed normocytic anemia (RBC 3.29 (4.7–6.1 M/*μ*L); MCV 82.1 (80.0–94.0 fL)) compatible with anemia of chronic disease. The white count was slightly elevated 14.5 (4.5–11.0 K/*μ*L) but normalized quickly after a short course of antibiotics. Surgery was consulted and a contrast enhanced CT of the chest was ordered to delineate the full extent of the mass and affected surrounding tissues. The CT revealed a massively large, heterogeneously enhancing, partially calcified, centrally necrotic mass immediately deep in the skin in the subcutaneous tissue of the thoracolumbar back region (Figure 1). This mass was centered more in the superficial tissue region without much involvement of the deeper structures. Incidentally, a chest plain film revealed a large double-density opacity overlying the cardiac silhouette on PA projection and was too posterior on the lateral projection to be visualized (Figure 2). This revealed that the mass was extrinsic to the bony thorax.

**Figure 3:**
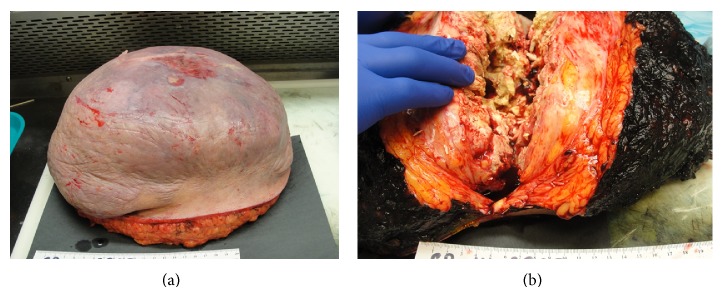
(a) The resected soft tissue mass from the back weighed 5634 grams. (b) Sectioning revealed a 24 cm well-circumscribed encapsulated firm mass centered in the dermis and extending to subcutaneous tissue. The mass demonstrated a central cavity with debris and pus.

**Figure 4:**
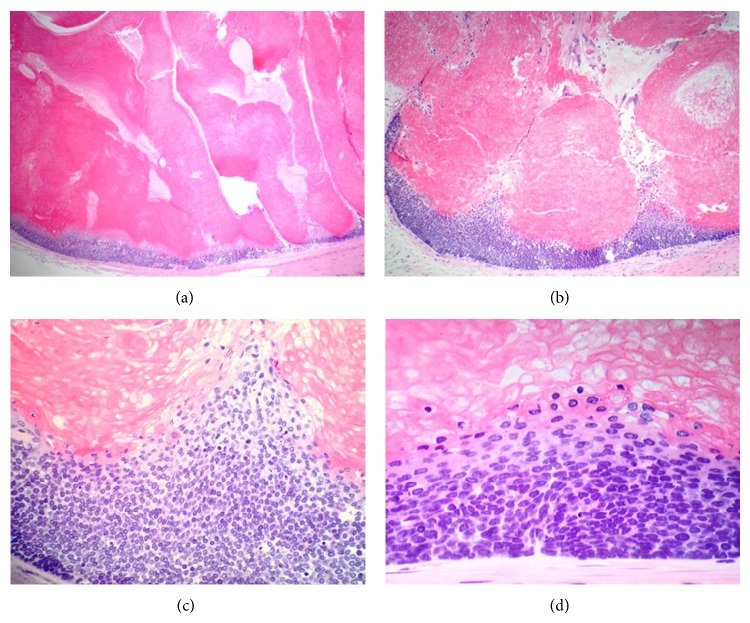
Histologic features of pilomatrixoma. ((a) and (b)) The tumor is composed of a biphasic population of basaloid and ghost cells. ((c) and (d)) Maturation of basaloid cells (bottom) into ghost cells (top). The cells become larger, acquire eosinophilic cytoplasm, and eventually lose their nuclei (H&E, ×40 (a), ×100 (b), ×400 (c), and ×600 (d)).

**Figure 5:**
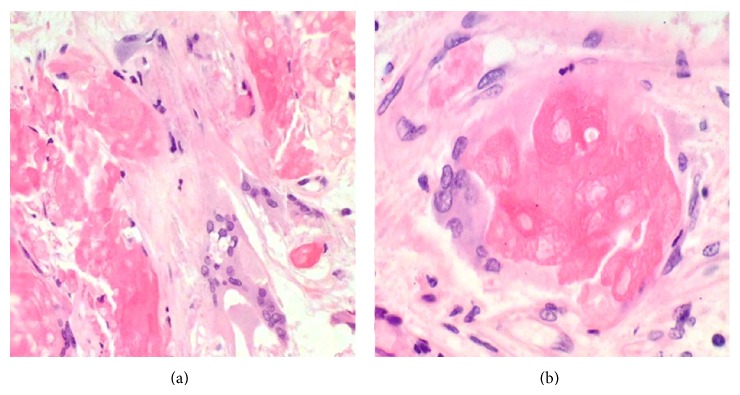
Foreign body giant cell reaction to the keratin (H&E, ×400 (a) and ×600 (b)).

Wide-excision surgery was performed and pathology revealed a massive soft tissue tumor of the back consistent with pilomatrixoma and with clean margins. The tumor measured 24 cm × 21 cm × 9 cm and was yellow-tan, well marginated, encapsulated, and firm. There was a cavity in the center of the tumor containing debris and pus (Figures 3(a) and 3(b)) consistent with central necrosis on CT examination. Histology from the resected tumor revealed the typical biphasic population of basaloid and ghost cells, which are characteristic of pilomatrixoma (Figures 4(a) and 4(b)). The basaloid cells mature to become the ghost cells (Figures 4(c) and 4(d)). Histologic sections also showed conspicuous multinucleate foreign body giant cell reaction, which is commonly present in this tumor type (Figures 5(a) and 5(b)). It is important to note that the tumor was extensively sampled and the histology was consistent with pilomatrixoma throughout, without any features of malignancy. Split-thickness skin grafts were used to close the wound and facilitate healing. The patient tolerated the surgery well and continues to keep regular follow-up visits with surgery.

## 2. Discussion

Pilomatrixoma was first termed as “calcified epithelioma of Malherbe” thought to originate from sebaceous glands by Malherbe and Chenatais in 1880. This was subsequently renamed by Forbis and Helwig in 1961 after demonstrating that its origin was actually from the hair follicle matrix cells [1, 2]. Pilomatrixoma is a benign neoplasm rarely exceeding 3 cm and typically affecting the head and neck regions, and rarely does it present in the extremities and it is even more rare in the trunk. The largest reported case was 7.5 cm [1, 3]. Our case is 24 cm, the largest to date.

Histologically, pilomatrixomas are nodular aggregates of epithelial cells scattered within a connective matrix with each nodule composed of two types of differently organized epithelial cells. The peripheral part is densely packed basophilic cells producing keratin; the central part contains eosinophilic cells known as “ghost” or “mummified” cells [4, 5]. Cytology from fine needle aspiration is an efficient first step in characterizing a lesion and often with consistent features but has limitations depending on predominance of a particular component over another [6, 7]. Malignant transformation is very rarely seen and associated with high rates of local invasion as well as metastasis [2].

The radiographic features of pilomatrixomas are very nonspecific findings regarding a soft tissue mass within the subcutaneous tissue demonstrating heterogeneity with variable enhancement and calcification on CT examination. Differential diagnosis would include malignancies such as malignant fibrous histiocytoma or synovial cell sarcoma versus benign tumors such as lipoma, fibromatosis, or hemangioma versus other causes like myositis ossificans, hematoma, or abscess [5, 8].

Pathology is the most definitive means of diagnosis with tissue sampling through either fine needle aspiration or open biopsy. CT would be helpful in surgical staging of malignant tumors or even large benign tumors as in our case.
